# Navigating thyroid hormone signaling in liver fibrosis: mechanisms and clinical implications

**DOI:** 10.1007/s40618-026-02820-8

**Published:** 2026-03-03

**Authors:** Cunsi Ye, Keyang Xu, Xiaopeng Lan, Shijian Quan, Yumeng Li, Wei Yang, Shuangquan Liu

**Affiliations:** 1 Department of Clinical Laboratory Medicine, Institution of Microbiology and Infectious Diseases, Hunan Province Clinical Research Center for Accurate Diagnosis and Treatment of High-incidence Sexually Transmitted Diseases, The First Affiliated Hospital, Hengyang Medical School, University of South China, Hengyang City, Hunan China; 2 Faculty of Medicine and Health, The University of New South Wales, Sydney, NSW Australia; 3 Faculty of Chinese Medicine, State Key Laboratory of Quality Research in Chinese Medicine, Macau University of Science and Technology, Macau, China; 4 School of Pharmaceutical Sciences, Guangzhou University of Chinese Medicine, Guangzhou, China

**Keywords:** Thyroid hormone signaling, Liver fibrosis mechanisms, Hepatic stellate cell activation, TGF-β/Smad and PI3K/AKT/mTOR signaling pathways, Therapeutic targets in fibrosis

## Abstract

**Background:**

Thyroid hormones have emerged as critical modulators of hepatic fibrogenesis, exerting regulatory effects through both genomic and non-genomic signaling pathways. Their influence spans multiple cellular processes, including the activation of hepatic stellate cells, metabolic regulation, inflammation, and extracellular matrix remodelling.This review aims to provide a comprehensive overview of the molecular mechanisms by which THs modulate liver fibrosis, and to explore the therapeutic potential of targeting TH-related signaling in clinical practice.

**Methods:**

We conducted an integrative narrative review of preclinical and clinical studies focusing on TH-mediated regulation of six key signaling pathways: TGF-β/Smad, PI3K/AKT/mTOR, Wnt/β-catenin, AMPK, NF-κB, and MAPK/ERK. Emphasis was particularly placed on their roles in HSC biology and fibrotic progression.

**Results:**

Although progress has been made in elucidating TH signaling in liver fibrosis, critical gaps remain—especially regarding downstream signal integration, pathway crosstalk, and human translational evidence. Preclinical studies consistently demonstrate that thyroid hormones exert antifibrotic effects by modulating metabolic and inflammatory pathways; however, clinical data supporting these findings remain limited. Evidence suggests that THs may exert antifibrotic effects via modulation of metabolic and inflammatory networks, and early investigations into THR agonists offer encouraging therapeutic prospects.

**Conclusions:**

Targeting thyroid hormone signaling represents a promising frontier in antifibrotic therapy. Clarifying mechanistic interactions and conducting well-designed clinical trials will be essential to translate these insights into effective interventions for patients with chronic liver disease.

## Introduction

### Definition and epidemiology of liver fibrosis

Liver fibrosis is a progressive pathological response to chronic or repeated injury, marked by excessive accumulation of collagen and other extracellular matrix (ECM) components that alter normal hepatic tissue architecture [1]. This fibrotic process is primarily driven by the activation of hepatic stellate cells (HSCs), which undergo transdifferentiation into myofibroblast-like cells, capable of secreting substantial amounts of collagen upon liver injury [2, 3]. The underlying mechanisms of liver fibrosis involve a complex network of inflammatory responses, cellular apoptosis, oxidative stress, and various signaling pathways that contribute to fibrosis progression [4].

Liver fibrosis is a key precursor to cirrhosis, with its progression laying the foundation for cirrhosis development, which is one of the leading causes of liver failure and liver-related mortality [5]. Early detection and intervention in liver fibrosis are critical for preventing its progression to cirrhosis and subsequent hepatic dysfunction. Liver fibrosis remarkably contributes to the rising incidence and mortality of liver diseases, making timely intervention essential, as both treatment efficacy and prognosis significantly decline once the disease progresses to cirrhosis. According to the Global Burden of Disease 2023 study, over two million individuals globally die from liver disease annually, including cirrhosis, viral hepatitis, and liver cancer, accounting for 4% of all deaths [6]. According to the WHO Global Health Estimates 2021, liver cirrhosis accounted for approximately 1.5 million deaths globally in 2021, ranking among the leading causes of liver-related mortality. These data underscore the critical role of liver fibrosis as a precursor to cirrhosis, which in turn significantly contributes to global liver failure and death rates [7]. Over recent decades, the mortality rates associated with cirrhosis have risen steadily, with hepatitis B and C infections being predominant etiologies in developing countries, while alcohol-induced liver disease and non-alcoholic fatty liver disease (NAFLD) are emerging as leading causes in high-income nations [8].

Recently, the nomenclature for fatty liver disease has undergone significant revision. The term NAFLD has been replaced by metabolic dysfunction–associated steatotic liver disease (MASLD), according to the international consensus, to better capture the metabolic abnormalities underlying this condition. This change reflects a conceptual shift from exclusion-based criteria toward recognition of the disease’s strong association with insulin resistance, obesity, and cardiometabolic disorders, which are key drivers of hepatic fibrosis [9].

The insidious progression of liver cirrhosis often leads to its diagnosis at a late stage, emphasizing the critical need for early screening and intervention. Notably, liver fibrosis, due to its subtle and often asymptomatic progression, is frequently diagnosed only after it has advanced to cirrhosis. This highlights the urgent necessity for early detection and intervention in liver fibrosis [10]. Therefore, understanding the early mechanisms of liver fibrosis and developing effective therapeutic strategies are vital for preventing its progression to cirrhosis and reducing cirrhosis-related mortality.

### Thyroid hormones in hepatic function

Thyroid hormones (THs), primarily consisting of thyroxine (T4) and triiodothyronine (T3), are crucial regulators of metabolic homeostasis. Secreted by the thyroid gland, these hormones exert widespread physiological effects, influencing cellular metabolism, growth, differentiation, and other vital bodily processes [11]. Through binding to specific nuclear receptors, thyroid hormones modulate gene transcription, thereby regulating metabolic pathways, cellular proliferation, and differentiation. They are essential for maintaining normal metabolic functions and facilitating proper growth and development [12]. Beyond their fundamental roles in metabolism, emerging research suggests that thyroid hormones also play pivotal roles in various pathological conditions, including metabolic syndrome, cardiovascular diseases, and liver disorders [13, 14]. In the liver, thyroid hormones regulate processes such as bile acid synthesis, lipid metabolism, and inflammation, all of which are integral to maintaining liver homeostasis. Moreover, recent studies indicate that thyroid hormones may influence liver fibrosis by modulating key processes such as stellate cell activation and ECM deposition, thus potentially affecting the progression of fibrotic changes in the liver [15]. Despite a growing body of evidence indicating that thyroid hormones may influence the onset and progression of liver fibrosis, the precise molecular mechanisms underlying this relationship remain unclear. A comprehensive understanding of these pathways is crucial for identifying novel therapeutic targets and advancing the field [12, 16, 17].

## Methods

This review was conducted through a comprehensive literature search aimed at summarizing current evidence on thyroid hormone signaling in liver fibrosis. Relevant studies were identified from PubMed, Web of Science, and Scopus databases, covering publications from January 2000 to October 2025. The search strategy combined keywords such as “thyroid hormone” “thyroid hormone receptor” “liver fibrosis” “hepatic stellate cell” “signaling pathway” and “fibrogenesis” Both preclinical (in vitro and animal models) and clinical (human studies) investigations were included. Additional sources were retrieved from the reference lists of key articles and recent systematic reviews.

Inclusion criteria comprised original research and review articles providing mechanistic, experimental, or translational insights into thyroid hormone–mediated signaling in hepatic fibrogenesis. Studies were excluded if they were non-English, conference abstracts without full data, or unrelated to liver pathology. The final selection prioritized studies with well-defined models, robust methodology, and relevance to thyroid hormone signaling in hepatic injury, fibrosis, and repair.

## Clinical insights of thyroid hormones

THs are essential regulators of systemic metabolism, maintaining basal metabolic rate, lipid and glucose turnover, and overall energy homeostasis. Dysregulation of TH signaling contributes to metabolic disorders such as diabetes, obesity, and metabolic syndrome [18] and altered TH levels have also been associated with cardiovascular abnormalities including hypertension, heart failure, and atherosclerosis [19]. In hepatic physiology, THs modulate lipid metabolism, oxidative stress, and inflammatory signaling, which are key processes underlying fibrogenesis [20]. Altered thyroid function can disrupt hepatic lipid balance and promote inflammation, thereby influencing the onset and progression of liver fibrosis [21].

Clinical studies have demonstrated a strong association between hypothyroidism and NAFLD, now redefined as MASLD [22]. Low circulating TH levels exacerbate hepatic steatosis and fibrosis risk, often accompanied by elevated liver enzyme levels that indicate hepatocellular injury [23]. Conversely, hyperthyroidism can lead to transient hepatic dysfunction, reflected by abnormal transaminases and histopathological injury [24].

While thyroid hormone replacement therapy can improve lipid metabolism and inflammatory profiles in hypothyroid patients, its role in directly treating liver fibrosis remains uncertain and is largely supported by preclinical evidence [25]. Early mechanistic studies suggested that T3 and its analogs prevent hepatic steatosis and inflammation, implicating thyroid hormones as potential modulators of metabolic liver disorders [26]. Consistent with these findings, activation of T3-dependent signaling has been shown to improve hepatic lipid metabolism and reduce steatosis in MASLD models [27]. More recently, the development of selective TRβ agonists, including resmetirom and VK2809, has shown promising results in improving hepatic fat content and metabolic parameters in patients with MASH, suggesting indirect benefits on fibrosis progression [28]. Collectively, these observations underscore the therapeutic potential of targeting the TH/TR axis to improve metabolic and inflammatory dysregulation in chronic liver disease, though further clinical validation is warranted [29, 30].

## Overview of thyroid signaling pathways in liver fibrosis

To elucidate the role of thyroid hormones in liver fibrosis, it is essential to first understand the key signaling pathways through which they mediate their biological effects in hepatic tissue. These pathways operate via both genomic and non-genomic mechanisms. Genomic actions are primarily mediated by thyroid hormone receptors (THRs), which act as nuclear transcription factors to regulate gene expression and will be discussed in detail in Sect. Thyroid Hormone Receptors and Genomic Signal Initiation and Nuclear Transcriptional Regulation in Genomic Signaling. In parallel, non-genomic signaling involves rapid, extranuclear events that influence intracellular signaling cascades, as outlined in Sect. Non-Genomic Signaling Pathways of Thyroid Hormones. Together, these mechanisms orchestrate a wide array of cellular processes, including lipid metabolism, oxidative stress, inflammation, and fibrogenesis—each playing a crucial role in the initiation and progression of liver fibrosis. A detailed overview of these pathways is provided in the following sections.

### Thyroid hormone receptors and genomic signal initiation

Thyroid hormones exert their effects through two specific nuclear receptors— thyroid hormone receptor alpha (TRα) and thyroid hormone receptor beta (TRβ)—members of the nuclear hormone receptor superfamily [31]. TRα is expressed mainly in the heart, skeletal muscle, and brain, maintaining cardiac contractility, thermogenesis, and neuromuscular function [32]. Although less abundant in the liver, TRα influences hepatic energy balance indirectly through systemic metabolic regulation.

In contrast, TRβ is highly expressed in metabolically active organs such as the liver, kidney, and thyroid gland [33]. In hepatic tissue, TRβ governs pathways involved in glucose utilization, lipid oxidation, and bile-acid synthesis [12, 34]. Dysregulation of TRβ signaling contributes to lipid accumulation, oxidative stress, and pro-inflammatory activation—key drivers of hepatic fibrogenesis [11, 35]. Collectively, TRβ serves as the primary mediator of thyroid hormone action in the liver, integrating systemic metabolic cues with transcriptional control. The following Sect. (Nuclear Transcriptional Regulation in Genomic Signaling) further details the genomic mechanisms through which TRβ regulates antifibrotic and metabolic gene networks relevant to hepatic fibrogenesis.

### Nuclear transcriptional regulation in genomic signaling

Building upon the receptor framework described above, TRβ-mediated genomic regulation constitutes the major mechanism through which THs exert long-term metabolic and antifibrotic effects [36]. This effect is especially significant in pathological conditions such as MASLD and metabolic-associated liver fibrosis, where thyroid hormone signaling is often impaired [37, 38]. Upon binding to T3, TRβ undergoes conformational change and forms heterodimers with retinoid X receptors (RXRs). These complexes bind to thyroid hormone response elements (TREs) in target-gene promoters, recruiting co-activators or releasing co-repressors to modulate gene transcription [39, 40]. In the liver, TH signaling enhances the expression of genes promoting fatty-acid oxidation (CPT1α, ACOX1) and mitochondrial biogenesis (PGC1α), while repressing profibrotic markers such as TGF-β1, α-SMA, and COL1A1, thereby favoring fibrosis resolution [41]. Experimental studies demonstrate that selective TRβ agonists (e.g., GC-1, VK2809) reduce hepatic collagen deposition, suppress HSCs activation, and improve liver histology, supporting the antifibrotic potential of TRβ-driven transcriptional programs [42].

Together, these findings identify TRβ as a pivotal regulator of hepatic metabolism and fibrogenesis, though the strength and direction of THR signaling may vary with disease stage and metabolic context, warranting further translational research.

### Non-genomic signaling pathways of thyroid hormones

In addition to their genomic effects, THs also exert significant influences through non-genomic mechanisms, facilitating rapid responses. These mechanisms operate independently of gene transcription, directly modulating intracellular signaling pathways. These non-genomic effects occur independently of gene transcription and involve rapid, membrane-associated interactions, typically through the activation of membrane receptors or other signaling molecules. This activation promptly triggers key intracellular signaling pathways, such as PI3K/AKT, AMPK, and NF-κB, initiating a cascade of downstream events that are integral to cellular metabolism, inflammation, and fibrosis [43].

A well-characterized mediator of these non-genomic effects is integrin αvβ3, a plasma membrane receptor that binds thyroid hormones and initiates downstream PI3K/AKT and MAPK (ERK1/2) signaling cascades. Activation of these pathways regulates cellular proliferation, survival, and angiogenesis, processes that are closely linked to hepatic inflammation and fibrogenesis. In hepatic tissues, integrin αvβ3–mediated thyroid hormone signaling may influence hepatocyte energy metabolism, inflammatory signaling, and the activation state of hepatic stellate cells, potentially modulating HSC proliferation, extracellular matrix deposition, and overall fibrogenic remodeling during liver fibrosis [39].

Moreover, the activation of G protein-coupled receptors (GPCRs) by thyroid hormones at the cell membrane. This interaction activates intracellular signaling cascades, such as the cyclic adenosine monophosphate (cAMP)-protein kinase A (PKA) pathway, which in turn regulates various cellular functions, including ion transport, enzyme activity, and metabolic processes [44].

**Fig. 1:**
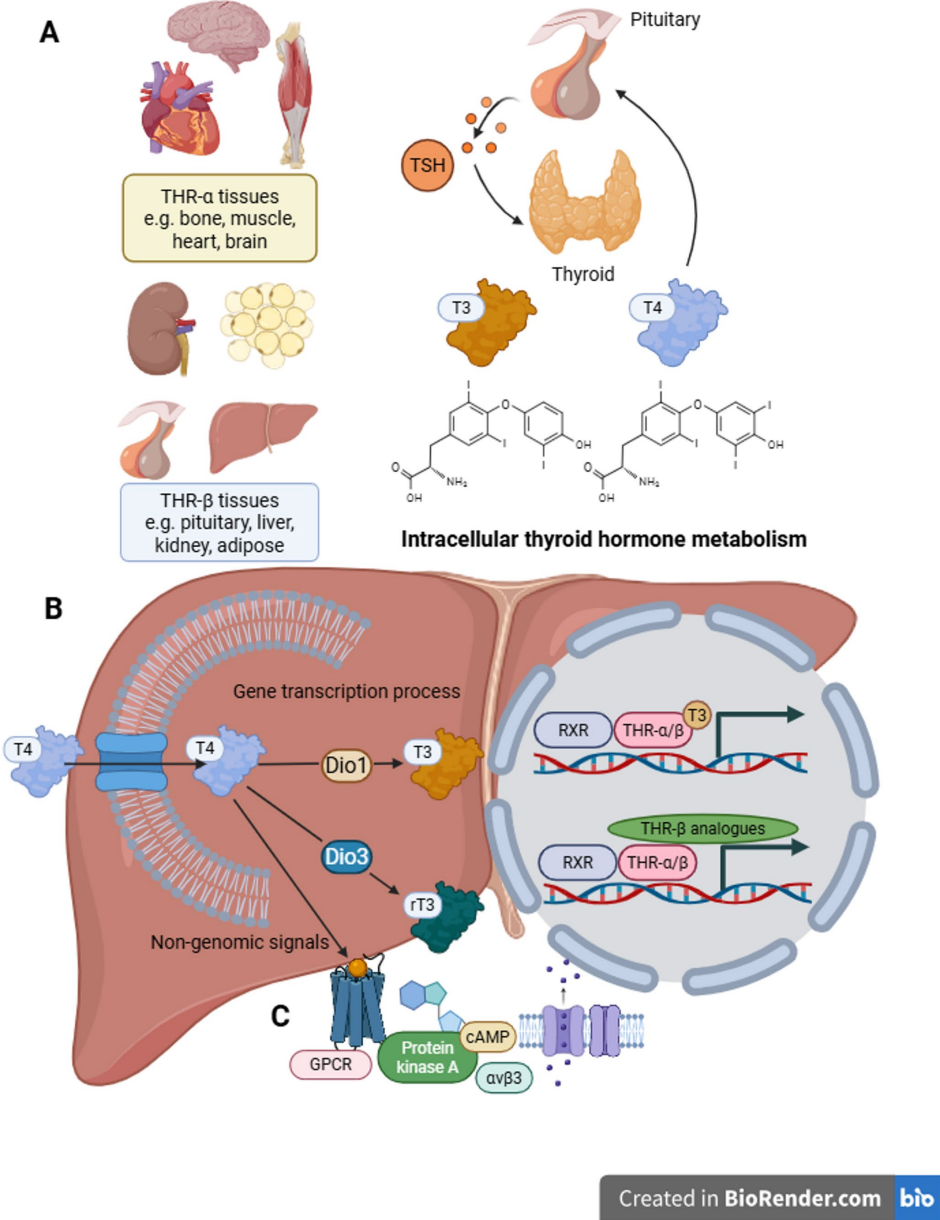
Thyroid hormone receptors and their signaling mechanisms in liver fibrosis. (A) Thyroid hormone receptors (TRα and TRβ) (B) Transcriptional Regulation (C) Non-Genomic Signaling Pathways

Taken together, non-genomic thyroid hormone signaling represents a crucial regulatory layer in liver pathophysiology. Through the TH–integrin–PI3K/AMPK and GPCR–PKA axes, THs rapidly remodel the intracellular environment of hepatocytes and HSCs, fine-tuning the balance between injury repair and fibrosis. Elucidating the interplay among these pathways will provide new insights into the multifaceted role of THs in liver fibrosis and may guide the development of THR-targeted antifibrotic therapies [45] (Fig. 1). 

## Regulation of liver fibrosis by thyroid hormones mediated various signaling pathways

Thyroid hormones modulate liver fibrosis not through a single linear cascade, but rather by engaging a network of interrelated signaling pathways that collectively influence HSC activation, ECM remodeling, and inflammatory responses. Understanding how thyroid hormone signaling intersects with these key fibrogenic pathways is essential for decoding its antifibrotic potential and identifying therapeutic intervention points. This section provides an in-depth examination of six major signaling pathways—TGF-β, PI3K/AKT/mTOR, Wnt/β-catenin, AMPK, NF-**κ**B, and MAPK/ERK—that are critically regulated by thyroid hormones and play pivotal roles in the development and resolution of liver fibrosis. Each pathway will be discussed in terms of its mechanistic contribution, crosstalk with thyroid hormone receptors, and potential clinical implications.

### TGF-β signaling pathway

The TGF-β signaling pathway plays a central role in the progression of liver fibrosis, primarily through the activation of downstream Smad transcription factors. Thyroid hormones, particularly T3, exert regulatory effects on this pathway by modulating the behavior of HSCs, which are key effector cells in fibrogenesis [46]. T3, acting via TRβ, has been shown to exert inhibitory effects on the TGF-β/Smad axis. Inhibition of TGF-βRI suppresses Smad phosphorylation in fibrotic mice, thereby reducing hepatic stellate cell activation and liver fibrosis [47]. Importantly, clinical studies have reported that patients with T2DM and MASLD often exhibit reduced serum levels of free T3 (FT3). These lower FT3 levels have been significantly correlated with the presence of advanced liver fibrosis, suggesting that thyroid hormones may serve not only as modulators of fibrogenic signaling but also as potential biomarkers for fibrosis severity. Collectively, these insights highlight the therapeutic potential of thyroid hormone signaling in counteracting the pro-fibrotic actions of the TGF-β pathway [48].

### PI3K/AKT/mTOR signaling pathway

The PI3K/AKT/mTOR signaling pathway is a central regulator of key cellular functions, including proliferation, survival, autophagy, and metabolic balance. It has been extensively implicated in the pathogenesis of liver fibrosis, particularly through its role in hepatic lipid accumulation and fibrogenic activation. In addition to the classical genomic actions mediated by nuclear thyroid hormone receptors, both T3 and T4 can modulate hepatic signaling pathways through non-genomic mechanisms involving the plasma membrane integrin αvβ3 receptor, which activates downstream PI3K/Akt and MAPK (ERK1/2) cascades. This integrin-mediated pathway has been characterized in preclinical models and is thought to link thyroid hormone signaling with processes such as cell proliferation, inflammation, and fibrogenesis [49].

Thyroid hormones are fundamental regulators of metabolic homeostasis, and accumulating evidence indicates their involvement in both fibrogenesis and broader metabolic liver disorders, such as MASLD [50]. Through their impact on hepatic lipid metabolism and insulin sensitivity, thyroid hormones modulate PI3K/AKT/mTOR signaling, which in turn affects lipid storage, oxidative stress, and inflammatory responses—key factors in fibrosis development [51, 52].

Mechanistically, thyroid hormones may activate or suppress components of the PI3K/AKT cascade in a context-dependent manner, thereby altering cellular energy status and fibrotic gene expression. This regulatory capacity underscores the therapeutic potential of targeting thyroid hormone signaling within this pathway. A deeper understanding of the molecular interplay between thyroid hormones and the PI3K/AKT/mTOR axis could pave the way for novel strategies to halt fibrotic progression and improve metabolic health in patients with liver disease [50].

### Wnt/β-catenin signaling pathway

The Wnt/β-catenin signaling pathway is crucial for regulating hepatocyte proliferation, differentiation, and regeneration, and its aberrant activation is strongly associated with fibrogenesis and hepatocarcinogenesis. In preclinical studies using human hepatoma cell models, THs, particularly T3, have been shown to suppress β-catenin signaling by upregulating the inhibitor Dickkopf-4 (DKK4), thereby reducing Wnt-driven proliferation and metastasis of hepatocytes. In rodent models of liver injury and hepatocellular carcinoma (HCC), activation of TRβ suppresses Wnt/β-catenin–dependent transcriptional targets, such as PDK1, while promoting PGC1α-mediated mitochondrial regulation that counteracts the metabolic reprogramming associated with fibrogenic and neoplastic progression [53]. These findings highlight a potential mechanistic link between endocrine regulation and fibrotic signaling, offering new insights into the complex interplay between thyroid function and hepatic fibrogenesis.

### AMPK signaling pathway

AMP-activated protein kinase (AMPK) is a master regulator of cellular energy homeostasis, responding to energy stress by modulating anabolic and catabolic pathways. In the liver, AMPK activation is essential for maintaining metabolic balance and protecting against fibrogenic stimuli. Thyroid hormones, particularly T3, influence AMPK signaling both by regulating upstream kinases and by directly modulating AMPK activity. Preclinical studies in Sprague-Dawley (SD) rat models have demonstrated that T3 enhances hepatic metabolic capacity by activating transcriptional programs that increase the expression of protective proteins, thereby improving cellular energy efficiency and stress resilience [54]. Mechanistically, in SD rat models, T3 promotes catabolic processes while downregulating anabolic pathways to boost ATP production, a metabolic shift that may influence fibrogenesis [55]. Importantly, in primary hepatic stellate cells models have demonstrated that AMPK activation has been shown to inhibit HSC activation and suppress collagen synthesis—two central features of liver fibrosis [56].These findings suggest that thyroid hormones, through modulation of AMPK signaling, may attenuate fibrotic progression by reprogramming hepatic energy metabolism and restraining profibrotic cell activation.

### NF-κB signaling pathway

The nuclear factor kappa-light-chain-enhancer of activated B cells (NF-κB) signaling pathway plays a pivotal role in regulating hepatic inflammation and fibrogenesis. Dysregulation of this pathway is closely associated with chronic liver injury and fibrotic progression. Emerging evidence from in vivo rat liver models and in vitro HSC-T6 hepatic stellate cell studies indicates that T3 can modulate NF-κB signaling, thereby influencing key pathological processes such as oxidative stress and apoptosis during liver fibrosis [57, 58]. In SD rat experimental models, T3 administration has been shown to enhance the hepatic expression of manganese superoxide dismutase (MnSOD) and B-cell lymphoma-2 (Bcl-2), both of which are involved in antioxidant defense and anti-apoptotic mechanisms [59]. This redox modulation is linked to the activation of Kupffer cells, which subsequently release tumor necrosis factor-alpha (TNF-α)—a cytokine that activates the IκB kinase (IKK) complex and initiates the NF-κB cascade. Through these actions, T3 supports hepatocellular resilience by promoting antioxidant enzyme production and preventing apoptosis. While NF-κB is often associated with pro-inflammatory signaling, its activation under thyroid hormone regulation may confer protective effects by enhancing the liver’s adaptive stress response [10]. These findings underscore the potential of targeting thyroid hormone–mediated NF-κB modulation as a therapeutic strategy against liver fibrosis.

### MAPK/ERK signaling pathway

**Fig. 2:**
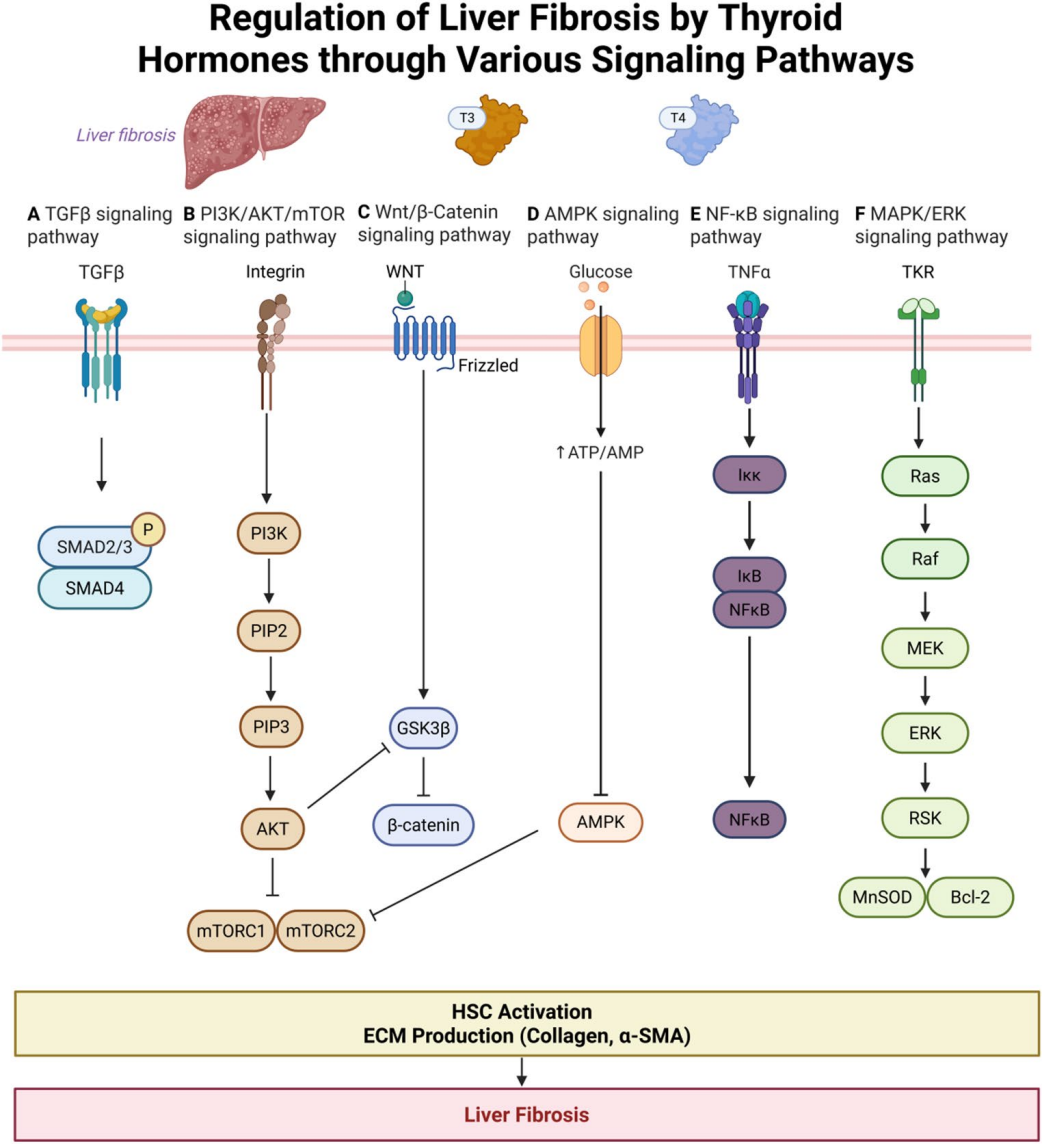
Regulation of Liver Fibrosis by Thyroid Hormones via Multiple Signaling Pathways. (**A**) TGF-β Signaling Pathway: T3 reduces TGF-β expression and Smad2/3 phosphorylation, inhibiting HSC activation. (**B**) PI3K/AKT/mTOR Signaling Pathway: THs modulate lipid metabolism and energy balance through this pathway. (**C**) Wnt/β-Catenin Signaling Pathway: THs influence HSC activation by regulating β-catenin stability and activity. (**D**) AMPK Signaling Pathway: T3 activates AMPK to suppress collagen deposition and maintain energy homeostasis. (**E**) NF-κB Signaling Pathway: T3 enhances antioxidant and anti-apoptotic responses via NF-κB modulation. (**F**) MAPK/ERK Signaling Pathway: T2 preserves ERK activity under metabolic stress, mitigating fibrosis progression

The mitogen-activated protein kinase/extracellular signal-regulated kinase (MAPK/ERK) signaling pathway is critically involved in cell proliferation, differentiation, and the pathogenesis of liver fibrosis. Thyroid hormones have been shown to influence this pathway, thereby modulating fibrotic progression [27]. Preclinical studies conducted in Wistar rat models have demonstrated that thyroid hormones, 3,5-diiodo-L-thyronine (T2) has emerged as a key modulator of the MAPK/ERK cascade. Experimental studies have demonstrated that T2 enhances hepatocyte proliferation and attenuates fibrosis by preserving ERK phosphorylation, even under metabolic stress conditions such as high-fat diet (HFD) exposure. This preservation of MAPK/ERK signaling integrity may protect against HFD-induced hepatic injury and fibrogenesis. These findings suggest that thyroid hormones, particularly T2, could exert antifibrotic effects through stabilization and modulation of the MAPK/ERK pathway. However, further research is needed to fully elucidate the underlying molecular mechanisms and to assess the therapeutic potential of targeting this axis in clinical settings [60] (Fig. 2). 

## Interaction and pathophysiological significance of signaling pathways

While individual signaling pathways contribute to distinct aspects of thyroid hormone regulation in liver fibrosis, these pathways rarely function in isolation. Instead, they engage in extensive crosstalk and functional integration, forming a complex regulatory network that governs hepatic fibrogenesis. Understanding these interactions is crucial, as the convergence of signaling inputs can amplify or attenuate fibrotic responses depending on the metabolic and inflammatory context.

Emerging evidence suggests that THRs function within a broader network of nuclear receptors that collectively regulate hepatic metabolism and fibrogenesis. In particular, THRs exhibit functional crosstalk with peroxisome proliferator-activated receptor γ (PPARγ), vitamin D receptor (VDR), and estrogen receptors (ERs), all of which have been implicated in liver fibrosis. Activation of THR-β and PPARγ promotes overlapping antifibrotic transcriptional programs that suppress hepatic stellate cell activation and collagen synthesis, while VDR cooperates with THR signaling to inhibit TGF-β/Smad-mediated profibrotic gene expression. Moreover, convergence between THR and ER pathways at the level of MAPK/ERK and PI3K/AKT cascades suggests integrated regulation of hepatocyte proliferation, inflammation, and metabolic remodeling. Together, these interactions highlight a coordinated nuclear receptor network through which thyroid hormone signaling may exert multifaceted control over hepatic fibrogenesis and repair [61].

This section explores two key dimensions of thyroid hormone-related signaling in liver fibrosis: (1) the dynamic interplay between major pathways—including TGF-β/Smad and PI3K/AKT/mTOR, Wnt/β-catenin and AMPK, as well as NF-κB and MAPK/ERK—and (2) the broader pathophysiological significance of these integrated networks in the context of liver injury and repair. Detailed information is provided in Sect. TGF-β Signaling Pathway–PI3K/AKT/mTOR Signaling Pathway.

### Interaction and integration of signaling pathways

Thyroid hormones regulate liver fibrosis through a variety of signaling pathways, which do not function in isolation but are interconnected, forming an intricate network that modulates the progression of liver fibrosis. The interactions among these signaling pathways play a pivotal role in shaping the overall regulatory effects on liver fibrosis and are crucial to understanding the multifaceted nature of hepatic pathophysiology [11].

#### TGF-β/Smad and PI3K/AKT/mTOR signaling pathway

Recent studies highlight intricate crosstalk between the TGF-β/Smad and PI3K/AKT/mTOR signaling pathways. Thyroid hormones, particularly T3, modulate the PI3K/AKT/mTOR pathway partly through the regulation of TGF-β expression and Smad protein activity [62]. A key mediator of this interaction is the MAPK cascade, which interfaces with Smad signaling to fine-tune HSC proliferation. Specifically, JNK facilitates cell proliferation, ERK is activated by platelet-derived growth factor (PDGF) to support mitogenesis, while p38 MAPK opposes this process by inhibiting HSC proliferation—thus maintaining a dynamic balance between pro- and anti-fibrotic signals. Further integration is observed between the TGF-β1/Smad7 axis and the NF-κB signaling pathway, especially in the context of liver inflammation. Loss of Smad7 amplifies NF-κB-driven inflammatory responses, leading to increased immune cell infiltration and heightened pro-inflammatory cytokine production. In contrast, Smad7 overexpression dampens these effects, likely via upregulation of the NF-κB inhibitor IκBα [63]. These interactions highlight the dual fibrotic and immunomodulatory roles of thyroid hormone-regulated signaling networks.

#### Wnt/β-catenin and AMPK signaling pathways

Thyroid hormones may modulate the Wnt/β-catenin signaling pathway indirectly via activation of AMPK, a key energy sensor that maintains metabolic homeostasis. AMPK plays a central role in regulating cellular energy status and exerts downstream effects on various signaling cascades, including Wnt/β-catenin. Emerging evidence suggests that thyroid hormones—particularly T3—can activate AMPK in hepatic cells, which in turn influences β-catenin stability and transcriptional activity [10]. The interplay between AMPK and Wnt signaling is context-dependent and may vary according to metabolic stress levels. In liver fibrosis, aberrant activation or suppression of either pathway has been associated with impaired hepatocyte function, increased fibrogenic activity, and metabolic dysregulation. These findings point to a complex regulatory axis in which thyroid hormones, via AMPK activation, influence the fibrogenic role of the Wnt/β-catenin pathway. This interaction underscores the broader metabolic-epigenetic integration underlying liver fibrosis and highlights potential therapeutic avenues for targeting thyroid hormone–linked signaling networks [64].

#### NF-κB and MAPK/ERK pathways

The NF-κB and MAPK/ERK signaling pathways exhibit a synergistic interaction that contributes significantly to hepatic inflammation and fibrosis. Activation of NF-κB has been shown to enhance MAPK/ERK signaling, thereby upregulating the expression of fibrogenic cytokines and promoting HSC activation [65, 66]. Aberrant co-activation of these pathways constitutes a critical pathological mechanism underlying chronic liver injury and fibrotic progression. Thyroid hormones, particularly T3, may influence this signaling axis by modulating NF-κB activity, which in turn affects ERK phosphorylation. Through this indirect regulation, thyroid hormones may either amplify or suppress fibrogenic signaling depending on the inflammatory context. This dual-level modulation underscores the intricate crosstalk between inflammatory and mitogenic pathways in liver fibrosis and highlights the potential for thyroid hormone signaling to serve as a therapeutic nexus in targeting multiple fibrogenic mechanisms.

### Pathophysiological significance

THs exert a profound influence on the pathophysiology of liver fibrosis through their interaction with multiple signaling pathways. These integrated networks not only govern HSCs activation but also modulate inflammatory responses and extracellular matrix remodeling—key features of fibrotic progression [20]. In the fibrotic liver, TH signaling intersects with major pathways such as NF-κB, MAPK/ERK, AMPK, and Wnt/β-catenin. When dysregulated, these interactions disrupt hepatocyte function, promote chronic inflammation, and accelerate collagen deposition. For instance, THs may exacerbate fibrosis by enhancing the expression of fibrogenic cytokines via the NF-κB and MAPK/ERK axes [66], while their influence on the AMPK pathway alters metabolic signaling, amplifying pro-inflammatory cues [67]. In parallel, TH-mediated stabilization of β-catenin may potentiate Wnt signaling, further driving fibrogenic activation [68]. This multifaceted interplay underscores the complex pathophysiological role of THs in liver fibrosis. Their regulatory influence extends beyond metabolic control, involving intricate signaling cascades that drive hepatocyte dysfunction, immune activation, and fibrotic remodeling [10]. Deciphering the crosstalk among TH-modulated pathways is critical to unraveling the molecular mechanisms underpinning fibrotic progression and identifying actionable therapeutic targets.

**Fig. 3:**
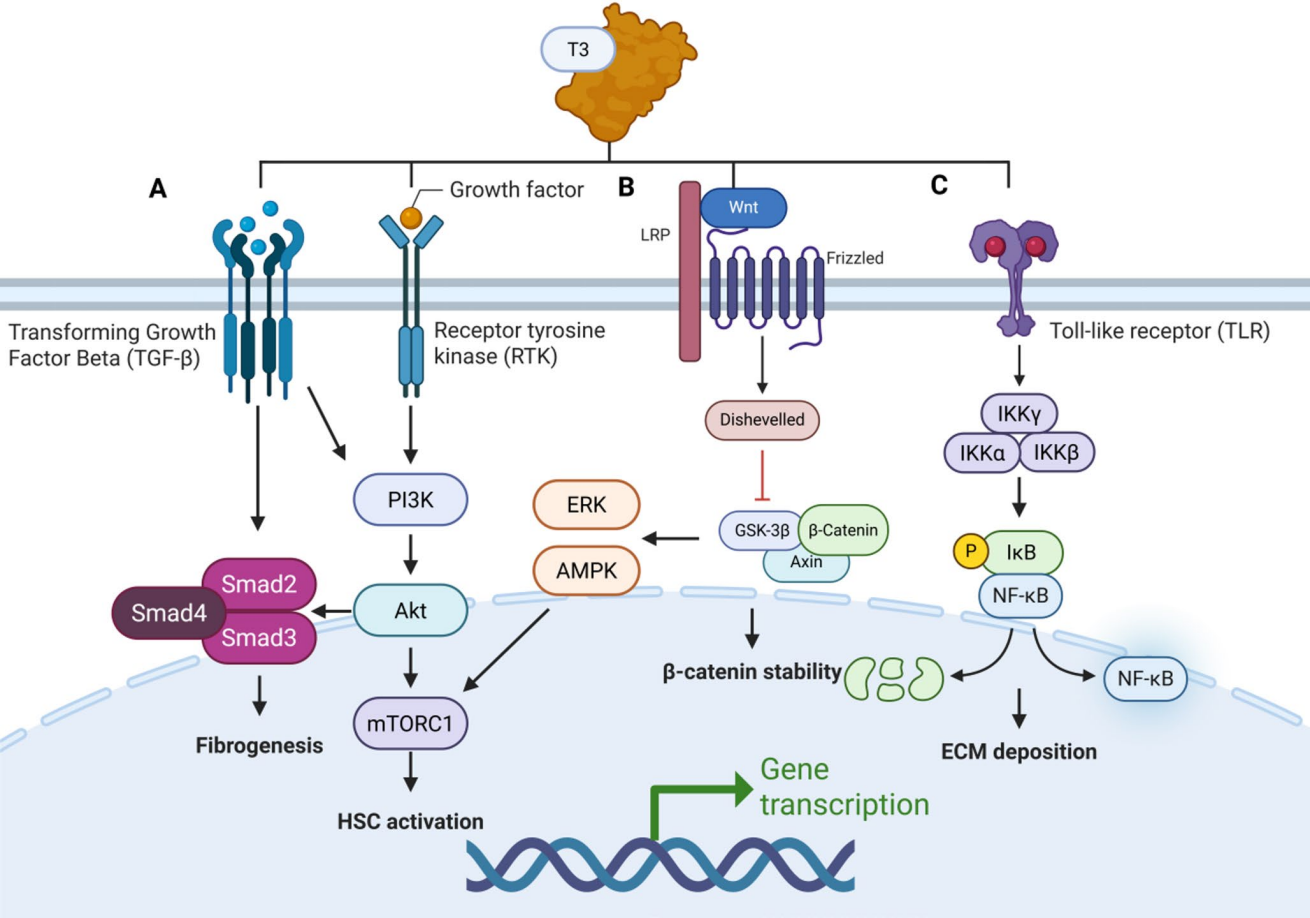
Interaction and Pathophysiological Significance of Signaling Pathways. (**A**) TGF-β/Smad and PI3K/AKT/mTOR Pathways: Crosstalk modulated by thyroid hormones affects HSC activation, with indirect input from MAPK signaling. (**B**) Wnt/β-Catenin and AMPK Pathways: THs regulate Wnt signaling via AMPK activation, influencing β-catenin stability and fibrogenesis. (**C**) NF-κB and MAPK/ERK Pathways: Synergistic interaction enhanced by THs promotes fibrogenic cytokine expression and fibrosis progression

From a clinical perspective, targeting components of TH signaling—such as NF-κB, MAPK/ERK, and Wnt/β-catenin—offers promising opportunities to attenuate hepatic inflammation and extracellular matrix deposition. Given the growing recognition of THs in metabolic regulation, their therapeutic relevance extends to related conditions, including MASLD and non-alcoholic steatohepatitis (NASH). Pharmacologic strategies aimed at restoring TH homeostasis or selectively modulating TH-related pathways may serve as valuable adjuncts to existing anti-fibrotic treatments. Importantly, the emerging role of TH signaling in liver pathology calls for well-designed clinical trials to evaluate the efficacy and safety of TH-targeted therapies. Such approaches could not only slow fibrosis progression but also improve outcomes in advanced liver diseases, including for patients undergoing transplantation. As our mechanistic understanding deepens, harnessing the therapeutic potential of thyroid hormone signaling may offer transformative advances in the management of liver fibrosis—a condition that remains largely irreversible and therapeutically challenging (Fig. 3). 

## Current challenges and future perspectives

### Challenges of thyroid hormone signaling pathways in liver fibrosis

Understanding the role of TH signaling in liver fibrosis is hindered by several critical challenges. These include the mechanistic intricacies of both genomic and non-genomic pathways, and the translational disconnect between experimental findings and clinical application. While considerable progress has been made in delineating THR-mediated regulation of HSC activation and liver inflammation, significant knowledge gaps persist. These gaps impede the development of targeted therapies and highlight the need for deeper mechanistic insight and more robust translational models [42, 69].

#### Intricacies of thyroid hormone signaling mechanisms

A fundamental challenge in elucidating TH signaling lies in the inherent complexity of its mechanisms. TH signaling encompasses both genomic and non-genomic pathways that collectively regulate key processes implicated in liver fibrosis, including cell survival, inflammation, and energy metabolism. For instance, the PI3K/AKT pathway mediates anti-apoptotic signaling, AMPK governs cellular energy balance, and NF-κB modulates [70, 71]. However, the precise contributions of these pathways to HSC activation and fibrotic matrix deposition remain insufficiently defined. Moreover, the context-dependent and overlapping activation of multiple signaling cascades adds an additional layer of complexity, making it difficult to dissect their individual roles in the fibrogenic process. This mechanistic ambiguity presents a major obstacle to the rational design of targeted interventions based on TH signaling.

#### Translational gaps in clinical research

One of the major barriers to translating TH signaling into clinical practice is the limited availability of long-term, high-quality clinical validation. Although the recent approval of resmetirom—a selective THR-β agonist—for MASH marks a promising step forward, the therapeutic efficacy of THR-targeted agents in liver fibrosis remains inadequately explored [72, 73]. A key limitation is the lack of robust clinical data assessing these interventions across diverse patient populations with varying fibrosis stages, metabolic statuses, and comorbidities. Bridging this translational gap necessitates well-designed clinical trials with rigorous patient stratification and long-term follow-up to ensure both efficacy and safety across real-world clinical settings.

#### Species-specific differences between animal models and human liver diseases

A critical limitation in liver fibrosis research is the translational gap arising from species-specific differences between animal models and human liver pathology. Although animal studies have provided valuable mechanistic insights into TH signaling, notable disparities exist in HSC activation markers, fibrosis resolution dynamics, and TH receptor isoform expression across species [42]. These interspecies differences can significantly affect therapeutic outcomes; for instance, THR-targeted agents that show efficacy in preclinical models often fail to replicate similar benefits in human trials. Such discrepancies highlight the pressing need for more physiologically relevant animal models that closely reflect human liver fibrosis, thereby improving the predictive validity of preclinical findings [74].

#### Technological and methodological limitations

The exploration of TH signaling in liver fibrosis is hindered by several technological and methodological constraints [75]. Although recent advances in single-cell transcriptomics and spatial proteomics have deepened our understanding of liver biology, current tools often lack the temporal resolution to capture real-time, dynamic interactions between TH signaling and other fibrogenic pathways such as Wnt and TGF-β [76]. This technological gap limits our capacity to dissect the integrated network of TH-regulated responses in vivo, making it difficult to fully elucidate the mechanistic underpinnings of TH-mediated liver fibrosis. Inadequate in vivo models and insufficiently sensitive detection techniques further hamper efforts to translate molecular insights into actionable therapies. Addressing these limitations will require a multidisciplinary approach that combines genomic, proteomic, and systems biology with translational and clinical research. Future investigations should prioritize the development of physiologically relevant animal models, implementation of real-time signaling analysis technologies, and well-structured clinical trials aimed at validating the therapeutic potential of TH receptor–targeted interventions [77].

### Future perspectives

Despite significant progress in elucidating the role of TH signaling in liver fibrosis, several key challenges remain unresolved. The duality of TH signaling—encompassing both genomic and non-genomic mechanisms—contributes to its biological complexity. The pleiotropic effects of THs on hepatocytes, HSCs, and immune populations are not yet fully understood, particularly in the context of their interplay with major fibrogenic pathways such as TGF-β, PI3K/AKT, and MAPK/ERK [11]. Most existing evidence is derived from animal models, raising concerns about translational applicability to human liver disease [66].

Mechanistic Insights: Advancing our understanding of TH signaling requires deeper mechanistic dissection of its interactions with fibrosis-associated pathways. High-resolution tools such as single-cell RNA sequencing and CRISPR-Cas9–based gene editing offer powerful platforms for studying TH-regulated processes at the cellular and molecular levels. These approaches will enable the identification of pathway-specific regulatory nodes and clarify how THs integrate with fibrotic signaling networks, thus informing the design of more targeted interventions [78].

Human Studies: To strengthen clinical relevance, large-scale cohort studies and well-controlled clinical trials are urgently needed. While animal models have illuminated basic mechanisms, human data are essential to validate the therapeutic potential of modulating TH signaling in liver fibrosis. Such studies will be instrumental in bridging the translational divide and guiding future therapeutic applications [79].

Targeted Therapies: Therapeutic exploration of THR agonists, TH analogs, or small-molecule modulators holds promise for anti-fibrotic intervention. Given the interconnected nature of fibrosis-associated signaling, combination strategies that target multiple pathways—including TH signaling—may yield superior efficacy compared to monotherapies. These multi-pronged approaches could offer more robust disease control, particularly in patients with chronic liver conditions [80].

In summary, thyroid hormone signaling has emerged as a critical regulator of liver fibrogenesis. However, realizing its full therapeutic potential requires a more comprehensive understanding of its mechanistic landscape and translational applicability. Future efforts should focus on refining preclinical models, leveraging cutting-edge molecular technologies, and conducting rigorous clinical validation to advance TH-based therapies for liver fibrosis and associated metabolic liver diseases.

## Conclusions

This review underscores the critical role of THs in the regulation of liver fibrosis, primarily through their modulation of key signaling pathways, including TGF-β/Smad, PI3K/AKT/mTOR, Wnt/β-catenin, AMPK, NF-κB, and MAPK/ERK. These pathways collectively orchestrate HSC activation, extracellular matrix remodeling, and fibrotic progression. The interconnected nature of these signaling networks reflects the multifactorial pathogenesis of liver fibrosis and highlights TH signaling as a promising target for therapeutic intervention.

Despite substantial mechanistic insights, significant gaps remain in fully elucidating the molecular interactions and translating preclinical findings into effective clinical strategies. A deeper understanding of TH-mediated signaling crosstalk—especially in human-relevant models—is essential for advancing precision therapies. Future efforts should prioritize the development of thyroid hormone analogs or pathway-specific modulators, along with rigorously designed clinical trials that incorporate patient heterogeneity.

In conclusion, targeting thyroid hormone signaling offers a compelling direction for the treatment of liver fibrosis, with the potential to reshape therapeutic paradigms for chronic liver diseases.
